# Making non-emergent abnormal results actionable: a short-term longitudinal qualitative study in two hospital-affiliated health examination centers in China

**DOI:** 10.3389/fpubh.2026.1900806

**Published:** 2026-08-19

**Authors:** Long Yao, Zhuojun Deng, Fuhua Luo, Lei He

**Affiliations:** 1 The First Affiliated Hospital of Shaoyang University, Shaoyang, Hunan, China; 2 The Second Affiliated Hospital of Shaoyang University, Shaoyang, Hunan, China

**Keywords:** follow-up behavior, health examination reports, health literacy, laboratory test results, organizational health literacy, qualitative research, risk communication, risk perception

## Abstract

**Background:**

Abnormal laboratory results in routine health examination reports can initiate prevention, confirmation, monitoring, or further consultation. Disclosure alone, however, does not make a result actionable: recipients must construct meaning, calibrate risk, and identify a feasible next step. This study examined non-emergent abnormal results as public health communication events in a regional hospital-affiliated health examination setting in China.

**Methods:**

A short-term longitudinal qualitative study was conducted with 48 adults who had received at least one non-emergent abnormal laboratory result at two health examination centers. Baseline interviews used participants' own de-identified reports and result vignettes as elicitation materials. A purposively selected subsample of 28 participants completed follow-up interviews 28–43 days later. Reflexive thematic analysis was the primary analytic methodology, supported by framework matrices for cross-case, site-level, and longitudinal comparison.

**Results:**

Five themes explained the result-to-action pathway: abnormality was noticed before it was understood; meaning was negotiated through biography, family, and social consequences; risk was alternately normalized and amplified; follow-up required navigation work; and participants described communication as more actionable when it was respectful, proportionate, time-bound, and linked to a service route. Follow-up trajectories included clinician-linked follow-up, planned monitoring, family or online advice without formal care by follow-up, self-directed repeat testing, and delay or no action. Clinician input or repeat results could feed back to reinterpret the original finding.

**Conclusion:**

Participants did not simply lack information; they encountered a health literacy environment that often transferred interpretive and navigation work to recipients and families. Health examination systems may support more proportionate and navigable follow-up by combining priority labels, plain-language meaning and uncertainty, time-bound next steps, trusted support, and recipient-controlled sharing. These communication components require clinical, usability, and implementation validation and should not be interpreted as clinical guidelines.

## Introduction

Health literacy concerns the knowledge, motivation, and competences required to access, understand, appraise, and apply health information for health care, disease prevention, and health promotion (1). Its public health importance lies not only in what individuals know but also in whether organizations create environments that make information understandable, proportionate, and usable (2, 3). In China, health literacy is an explicit health-promotion priority (4), and recent validation of a simplified Chinese clear-communication index emphasizes main messages, behavioral recommendations, numbers, and risk explanation (5). Limited health literacy is associated with poorer outcomes through pathways involving access, communication, and self-care (6, 7). Routine laboratory reports are therefore not neutral data displays; they are organizational communication environments.

Routine health examinations seek to identify early metabolic, hepatic, renal, infectious, endocrine, and other risk signals before symptoms become severe. Yet an out-of-range value rarely speaks for itself. Interpretation requires numeracy, terminology, biological variability, reference ranges, and uncertainty. Numeracy shapes how people compare values with thresholds (8, 9), while risk communication is strengthened by plain language, meaningful categories, and formats that support deliberation rather than fear (10, 11). Risk perception is also affective and social: familiarity can attenuate concern, whereas anxiety, prior illness experience, family narratives, and online search can amplify it (12, 13).

Patient-safety research has documented failures to communicate and follow up abnormal test results in ambulatory care (14–17), and laboratory medicine has emphasized that testing creates value only when results inform decisions and avoid harm (18–20). Research on patient-facing laboratory information shows persistent difficulty identifying out-of-range values, interpreting reference ranges, and deciding whether action is needed (21–26). Recent studies further show that portal access does not reliably eliminate comprehension problems, that graphs may produce false calm or false alarm, that repeated portal access can signal worry and messaging burden, and that movement from access to activation depends on interpretive support, emotion, workflow, and equity (27–30). Access is therefore not interpretation, and interpretation is not action.

This communication problem is visible in hospital-affiliated health examinations in China, where results may be delivered through hospital applications, WeChat-linked mini-programs (lightweight applications embedded within WeChat, a widely used Chinese multipurpose messaging and mobile-services platform), printed reports, or combinations of these formats. Recipients may have to choose a department before professional triage and may need to decide whether to return to a tertiary hospital, use local services, seek family help, or wait. In this study, actionability refers to the extent to which result communication enables a recipient to identify a proportionate, time-bound, and navigable next step while preserving diagnostic uncertainty. Actionability is distinct from action, clinical appropriateness, and eventual outcome.

The study asked: how do adults receiving non-emergent abnormal laboratory results in health examination reports understand the results, appraise risk, and decide whether and how to seek follow-up? Subsidiary questions examined visual flags and reference ranges; family, peer, and online mediation; barriers to timing and service navigation; and the communication features participants considered credible, respectful, and usable. The objective was not to evaluate laboratory accuracy, test-order appropriateness, or clinical outcomes, but to explain the communication mechanisms through which abnormal results became different short-term trajectories in two hospital-affiliated centers in one region of China.

## Materials and methods

### Study design and reporting

A short-term longitudinal qualitative design was used because the phenomenon of interest was not a single decision at report receipt but a sequence of interpretation, risk appraisal, navigation, and response over time. Baseline interviews were conducted within 30 days of report receipt; follow-up interviews examined how initial intentions changed 28–43 days later. This design supported comparison of immediate interpretation with subsequent participant-reported trajectories and identification of points at which concern was redirected, delayed, or converted into a feasible plan (31).

Reporting was guided by the Consolidated Criteria for Reporting Qualitative Research and the Standards for Reporting Qualitative Research (32, 33). The study classified reported trajectories but did not independently adjudicate their clinical appropriateness. Accordingly, terms such as planned monitoring, self-directed repeat testing, and clinician-linked follow-up describe participants' accounts and their alignment with a stated report or clinician recommendation; they are not clinical judgments about the optimality of care.

### Conceptual orientation

Health literacy, organizational health literacy responsiveness, risk perception, and test-result follow-up were used as sensitizing concepts rather than fixed coding categories. Health literacy oriented attention to access, understanding, appraisal, and application (1–7). Organizational responsiveness directed attention to whether report design, staff explanations, mobile interfaces, and appointment systems reduced or transferred complexity to recipients (34, 35). Risk-perception concepts sensitized the analysis to affect, uncertainty, severity, susceptibility, familiarity, and social comparison (12, 13). Test-result follow-up literature focused attention on the practical path from notification to a service-linked response (14–20).

The analytic premise was that information transfer alone does not create actionability. A result must be noticed, interpreted, calibrated as a proportionate concern, linked to a feasible route, and translated into a time-bound next step. The analysis also tested non-linear cases in which family or online information repeatedly changed meaning, service barriers redirected participants back to information seeking, and repeat testing or clinician input revised the initial risk appraisal.

### Setting

The study was conducted between September 2025 and March 2026 at two hospital-affiliated health examination centers in one prefecture-level region of China. Site A contributed 27 baseline participants and 16 follow-up participants; Site B contributed 21 baseline participants and 12 follow-up participants. Both centers served urban districts, county towns, and surrounding townships and issued reports containing routine blood, urine, biochemical, immunological, and selected disease-specific test results. Non-emergent results were operationalized as abnormalities that did not trigger either center's critical-result notification or emergency-referral protocol at report release. Results that triggered either process were excluded and remained subject to each hospital's established escalation procedures.

Site A primarily released verified results through a hospital application, with printed copies available on request. Site B commonly used a WeChat-linked mini-program together with routine staff printouts. Both formats displayed values, reference ranges, and abnormality flags, but the amount of plain-language interpretation and department guidance varied. A structured site-level comparison found the five themes at both centers; differences in release channel and staff handover shaped examples of visibility and navigation but did not create site-specific themes.

Report access was distinguished from digitally mediated navigation capacity. Report access referred to whether the participant received paper, electronic, or both formats. Digitally mediated navigation capacity referred to the reported ability to open an electronic report independently, follow official links, and complete online registration. The study used report access as a descriptive characteristic and treated digital capacity as contextual qualitative information rather than a quantified literacy score.

### Participants and sampling

Participants were eligible if they were aged 18 years or older, had received a health examination report within the previous 30 days, had at least one non-emergent abnormal laboratory result as defined above, and could participate in a Mandarin-language interview. Purposeful maximum-variation sampling sought diversity in age, sex, education, residence, result cluster, report format, digital context, and initial follow-up intention. Low-frequency result clusters were included to broaden analytic contrast, not to support result-specific subgroup claims.

Recruitment was conducted through center staff, who provided a study information sheet after report release. Interested adults contacted the research team directly, and center staff were not told who accepted or declined. 48 adults completed baseline interviews. After review of structured baseline summaries, Long Yao and Zhuojun Deng jointly selected 32 participants for purposive follow-up before the final thematic structure was fixed. Selection sought variation in baseline reaction, result cluster, residence, report access, and initial intention.

Twenty-eight of the 32 invited participants completed follow-up; two declined because of time constraints and two could not be reached after three contact attempts. Follow-up occurred a median of 34 days after baseline (range 28–43 days). The flow comprised 48 baseline participants, 32 participants purposively invited to follow-up, and 28 participants who completed follow-up. Baseline and follow-up subsample characteristics are presented side by side in Table 1.

**Table 1:** Baseline participant characteristics and purposive follow-up subsample profile.

Characteristic	Category	Baseline, *n* = 48	Follow-up, *n* = 28
Study site	Site A	27	16
Study site	Site B	21	12
Sex	Female	27	15
Sex	Male	21	13
Age group	18–34 years	8	4
Age group	35–49 years	14	9
Age group	50–64 years	17	10
Age group	≥ 65 years	9	5
Residence	Urban district	15	11
Residence	County town	16	11
Residence	Township/rural village	17	6
Education	Lower secondary or less	8	5
Education	High school/technical school	20	13
Education	College	14	6
Education	Postgraduate	6	4
Result cluster	Liver enzyme abnormality	10	5
Result cluster	Kidney/urine abnormality	8	4
Result cluster	Multiple mild metabolic abnormalities	9	4
Result cluster	Infectious serology ambiguity	6	3
Result cluster	Glycemic abnormality	6	5
Result cluster	Thyroid marker abnormality	5	3
Result cluster	Lipid abnormality	3	3
Result cluster	Tumor-marker borderline elevation	1	1
Report access	Paper only or staff printout	17	11
Report access	Mobile/electronic report	22	12
Report access	Both paper and electronic	9	5
Baseline intention	Prompt consultation or repeat test	21	13
Baseline intention	Lifestyle change or delayed review	15	9
Baseline intention	Uncertain/no clear plan	12	6

Counts are descriptive. The follow-up subsample was purposively selected and should not be treated as a representative attrition sample. Low-frequency result clusters were retained for analytic contrast rather than subgroup inference.

Sampling was guided by information power rather than a fixed sample-size rule (36). The question was specific, participants had recent first-hand experience, interviews were report-anchored, and the sample contained variation relevant to actionability. After approximately 40 baseline and 22 follow-up interviews, later data mainly refined boundary conditions, site contrasts, and temporal pathways rather than altering the thematic structure. The final interviews were used to challenge candidate themes and the recursive model.

### Data collection

Baseline interviews were semi-structured and used report elicitation. Participants were invited to show a de-identified section of their own report and describe what they noticed, what the result meant, how serious or urgent it seemed, what information they sought, who they consulted, and what next step appeared feasible. Short result vignettes were also used to explore communication preferences beyond a participant's own result. Vignettes informed coding and comparison, but no direct quotation in the final Results section was drawn solely from a vignette response.

Follow-up interviews examined actions taken, changes in risk perception, reasons for action or delay, repeat testing, additional information seeking, and retrospective views of the original report. The dominant trajectory was classified as clinician-linked follow-up; planned monitoring; family or online advice without formal care by the follow-up interview; self-directed repeat testing without professional interpretation; or delay or no action by the follow-up interview. Classification was descriptive and based on the interview narrative, not on independent clinical adjudication.

Baseline intention was classified as prompt consultation or repeat testing, lifestyle change or delayed review, or uncertain/no clear plan. Prompt consultation or repeat testing denoted an intended clinician contact or repeat test as the next step; lifestyle change or delayed review denoted a stated behavioral change or deferred review with some intended follow-up; and uncertain/no clear plan denoted no identifiable next action. When participants expressed more than one intention, the category representing their most immediate intended next step was used.

Long Yao and Zhuojun Deng conducted the interviews. Both interviewers had training in qualitative interviewing and backgrounds in public health and health-services research. Neither worked at the examination centers, provided clinical care to participants, nor had a prior relationship with them. Baseline interviews included 36 in-person sessions, 8 secure video sessions, and 4 telephone sessions; follow-up interviews included 15 in-person, 7 secure video, and 6 telephone sessions. A family member was present at three baseline and two follow-up interviews at the participant's request. Family comments were documented in field notes but were not coded as participant data unless the participant explicitly adopted the information in their own account. Baseline interviews lasted 38–82 min and follow-up interviews 18–38 min. All were audio-recorded with consent, transcribed verbatim in Mandarin, and de-identified.

Vignette wording and sample communication language were reviewed before field use by one laboratory medicine physician and one primary care clinician to reduce clinical overreach. The vignettes were elicitation tools, not diagnostic advice. Any interval mentioned by a participant was analyzed as an example of desired time-boundedness unless it had been explicitly reported as a clinician's recommendation.

### Quotation translation and source classification

All quotations were translated from Mandarin Chinese into English by Long Yao and independently checked by Zhuojun Deng against the original transcripts. Differences were resolved through discussion to preserve substantive meaning, tone, hesitation, and uncertainty. Minor grammatical smoothing was permitted only when it did not alter meaning. The internal quote audit recorded whether each excerpt came from baseline or follow-up, whether it concerned the participant's own report, its transcript location, result cluster, final manuscript use, and follow-up category.

### Data analysis

Analysis was conducted in Mandarin Chinese. Reflexive thematic analysis was the primary methodology (37–39). Framework matrices were used as a secondary organizing tool for cross-case, site-level, and longitudinal comparison rather than as a coding-reliability procedure (40). De-identified transcripts and separately stored audio recordings were kept in encrypted, access-controlled project folders; code and case matrices were maintained in Microsoft Excel 365.

The analytic team read transcripts and field notes repeatedly. Initial coding stayed close to participant language, including ‘arrow means abnormal but not urgency,' ‘family as translator,' ‘commonness as safety,' ‘search before choosing a department,' ‘recheck without when,' ‘not emergency but not nothing,' and ‘repeat test to calm myself.' Differences between analysts were used to identify assumptions, refine codes, and strengthen interpretation. Analytic meetings occurred after the first 10 interviews, after 25 interviews, during early follow-up, and after assembly of the complete dataset.

Framework matrices compared age group, education, residence, site, result cluster, report access, digital context, initial intention, and follow-up trajectory. Education was retained as a descriptive cross-case dimension but was not used to assign causal meaning to individual case quotations. The matrices distinguished recognition from actionability, planned monitoring from unstructured delay, family-mediated navigation from dependency, and self-directed repeat testing from clinician-linked interpretation.

Long Yao assigned preliminary baseline-intention and follow-up-trajectory categories from paired case summaries, and Zhuojun Deng checked all 28 classifications against the paired interview narratives. Differences were resolved through discussion. Planned monitoring required an explicit intended time or trigger and a feasible route; otherwise, absence of action by follow-up was classified as delay or no action.

Candidate themes were developed by asking what cognitive, social, organizational, and communicative work was required for an abnormal result to become a navigable next step. Discrepant cases included participants who remained uncertain despite strong information-seeking skills, participants who acted after a family member created a concrete plan, participants who understood concern but lacked a service route, and participants whose repeat testing produced temporary reassurance without interpretation. Site-level comparison and follow-up accounts were used to test the boundaries of each theme.

Every final direct quotation and every shortened table excerpt was checked against the de-identified transcript, participant master record, result cluster, source interview, and follow-up category. The complete participant-level master record and quote audit are retained as confidential study documentation and are not publicly released because combinations of residence, result type, and trajectory could increase re-identification risk in this regional two-center sample.

### Reflexivity and trustworthiness

The author team recognized that public health researchers may assume that clearer explanation automatically produces more action. Reflexive memos therefore examined alternative interpretations, including the possibility that a participant's planned monitoring reflected constraints or prior advice rather than disengagement, and that self-directed repeat testing could be a pragmatic strategy while still failing to create understanding. The team explicitly separated the actionability of communication from clinical appropriateness.

Trustworthiness was supported through maximum-variation sampling, short-term longitudinal follow-up, report elicitation, field notes, team memoing, discrepant-case analysis, site comparison, complete quote audit, and an audit trail linking data segments to codes, themes, and model components (41, 42). A concise findings summary was discussed with six participants and four center staff members. Feedback strengthened the emphasis on time-bound next steps, the distinction between reassurance and dismissal, and the description of family members as translators, navigators, amplifiers, and risk-sharing partners.

### Ethical considerations

Ethical approval was granted by the Ethics Committee of the First Affiliated Hospital of Shaoyang University (approval no. 20250810001), with site authorization from the Research Ethics Office of the Second Affiliated Hospital of Shaoyang University (authorization no. 20250812001). All participants provided written informed consent for baseline interviews, use of de-identified quotations with limited analytic descriptors, and separate follow-up contact. Participants were reminded that the study did not provide diagnosis or treatment advice. Data were de-identified, and family-oriented communication implications were interpreted as recipient-controlled sharing rather than automatic disclosure to relatives.

## Results

48 participants completed baseline interviews, and 28 of 32 purposively invited participants completed follow-up. The side-by-side profile in Table 1 shows that the follow-up subsample retained variation in site, sex, age, residence, education, result cluster, report access, and baseline intention. Because follow-up sampling was purposive, the table is descriptive and is not used for population inference.

The analysis generated five themes. Their mechanisms, evidence texture, boundary conditions, and temporal implications are summarized in Table 2. The full descriptive transition matrix for all 28 follow-up participants is shown in Table 3. The matrix demonstrates that the same baseline intention could lead to clinician-linked follow-up, planned monitoring, family or online advice without formal care by follow-up, self-directed repeat testing, or delay or no action. Percentages and statistical tests were not used because the follow-up subsample was purposively selected. The paired accounts reported below show how selected transitions unfolded over time.

**Table 2:** Themes, mechanisms, evidence texture, and analytic boundaries.

Theme	Mechanism	Exemplar evidence texture	Boundary/longitudinal implication
Abnormality as signal before meaning	Visual flags attracted attention but did not communicate severity, temporality, priority, or action.	‘Red for a small thing and red for a big thing looked the same' (P26).	Repeated exposure could produce desensitization; concern without priority could become delay/no action.
Meaning negotiated in everyday networks	Biography, family, prior illness, and social consequences shaped interpretation.	‘Creatinine was just a word. My daughter said it involved the kidney' (P28).	Family could translate, normalize, amplify, or share risk; sharing should remain recipient-controlled.
Risk between normalization and amplification	Common findings were normalized; ambiguous findings were amplified by online or social information.	‘Familiar became safe, even though I knew safe and common are not the same thing' (P12).	Information volume did not guarantee calibration; trusted context and boundaries around uncertainty were needed.
Follow-up as navigation work	A response required knowing when, where, and through which service to seek advice or repeat testing.	‘Recheck without a time is like no instruction' (P07).	Planned monitoring differed from unstructured delay; self-directed repeat testing could reassure without interpretation.
Respectful actionability	Clarity worked when it preserved dignity and linked meaning, uncertainty, timing, and route.	‘It became a task, not a fear' (P35).	The study identified communication preferences, not clinically validated intervals or interventions.

Shortened excerpts are drawn from final audited quotations. The study describes participant-reported trajectories and communication conditions; it does not adjudicate clinical appropriateness.

**Table 3:** Descriptive transition matrix from baseline intention to short-term participant-reported trajectory.

Baseline intention	Clinician-linked follow-up	Planned monitoring	Family/online advice without formal care	Self-directed repeat testing	Delay/no action	Row total
Prompt consultation or repeat test	7	2	1	2	1	13
Lifestyle change or delayed review	2	4	1	1	1	9
Uncertain/no clear plan	1	0	2	1	2	6
**Column total**	**10**	**6**	**4**	**4**	**4**	**28**

The matrix displays all 28 follow-up participants as mutually exclusive dominant trajectories. Baseline-intention and follow-up-trajectory categories were assigned and checked as described in Methods. Planned monitoring required an explicit intended time or trigger and a feasible route; otherwise, absence of action was classified as delay/no action. The family/online-advice category indicates that no formal care had occurred by the follow-up interview. Because follow-up was purposively selected, no percentages, statistical tests, or population estimates are reported. Bold values indicate column totals, including the overall total.

### Theme 1: Abnormality was read first as a signal before it became meaningful health information

Participants generally noticed an abnormality marker before they understood the laboratory value. Arrows, red values, bold type, and the word ‘abnormal' attracted attention, but a visual signal answered only ‘is something different?' It did not establish seriousness, temporality, priority, or the first next step.

A county-town participant described this gap: ‘The report said an arrow was high, but it did not tell me whether this was dangerous today or something to watch. I knew the arrow meant abnormal, but not what to do before it became diabetes' (P03, female, 35–49 years, county town, glycemic abnormality). Another participant looking at a mobile report said, ‘It was red, yes. But red for a small thing and red for a big thing looked the same to me' (P26, male, 50–64 years, county town, thyroid marker abnormality).

When several mild abnormalities appeared together, equal visual treatment could make them seem equally important. One participant said, ‘Six arrows made me feel old overnight. Later one doctor said none was urgent, but the report had already made all six look equal' (P05, female, 50–64 years, county town, multiple mild metabolic abnormalities). Mobile display could add urgency without calibration; P18 described how a red HbA1c value increased family concern even though the interface did not explain priority.

Familiarity created the opposite boundary condition. P23 said, ‘Every year my report has arrows. I looked first, then closed it. If every arrow looks the same, after a few years none looks important' (male, 35–49 years, township/rural village, lipid abnormality). At follow-up, concern without priority sometimes became no action, whereas a brief handwritten indication of which item to address first converted a less dramatic visual reaction into a concrete plan. Visual salience was therefore necessary but insufficient.

### Theme 2: Meaning was negotiated through family, biography, and the social consequences of labels

Participants interpreted results through family histories, prior illness, workplace talk, memories of relatives' diagnoses, and anticipated social consequences. Terms such as ‘positive', ‘weakly positive,' ‘marker,' and ‘antibody' could carry implications about contagion, stigma, family responsibility, or hidden disease that were not addressed by reference ranges.

A participant with ambiguous infectious serology explained: ‘The words positive and weakly positive are not small words for a family. I wanted to know whether I could eat with my grandson, not only whether the laboratory result was technically correct' (P40, female, 35–49 years, county town). Family discussion could create either understanding or shared uncertainty.

Family members often acted as translators and navigators. A rural older adult said, ‘Creatinine was just a word. My daughter said it involved the kidney, so I listened to her, not the report. Without her, the paper would have gone into a drawer' (P28, female, ≥65 years, township/rural village, kidney/urine abnormality). Another participant deliberately asked an older sibling to judge the route with her because choosing the wrong department would waste a full day (P29, female, 35–49 years, township/rural village, multiple mild metabolic abnormalities). This was shared uncertainty management, not simply compensation for an individual deficit.

Family mediation could also normalize or amplify concern. P11 received conflicting messages about thyroid antibodies from a relative and a colleague. P41 understood that urine protein was outside the reference range but not whether it was temporary or persistent; at follow-up, her daughter had arranged a local morning-urine repeat. Meaning therefore emerged through negotiation among report language, biography, social stakes, and the practical capacities of trusted others.

### Theme 3: Risk perception oscillated between normalization and amplification

Common abnormalities such as high lipids, uric acid, mild glucose elevation, or liver-enzyme changes were sometimes minimized because participants knew many people with similar results. P12 stated, ‘Because everyone in the office has high lipids, it felt normal. Familiar became safe, even though I knew safe and common are not the same thing' (male, 35–49 years, urban district, lipid abnormality).

Ambiguous results produced the opposite pattern. Participants searched because reports lacked context for urgency or department choice, and search results exposed them to severe possibilities that were difficult to calibrate. P17 said, ‘I was not scared by the number itself; I was scared because the internet gave 10 possible diseases. One page said fatty liver, another said cancer, and I could not tell what matched my report' (male, 50–64 years, urban district, liver enzyme abnormality).

The most unstable appraisals occurred when social advice conflicted with professional advice. P46, whose borderline tumor marker was used only as an analytic contrast, described relatives recommending immediate computed tomography while a clinician recommended repeat testing. The case illustrates uncertainty and social amplification, not a claim about all tumor-marker findings.

A contrasting pathway emerged when family support converted uncertainty into a simple plan. P43 recognized that the HbA1c result was abnormal but did not understand the abbreviation; her son wrote ‘morning fasting, community clinic, next Tuesday.' This case is revisited in the paired longitudinal analysis below.

### Theme 4: Follow-up was navigation work, not only motivation

Participants often intended to respond but did not know which department to choose, whether a local repeat test was sufficient, whether fasting was required, how soon to act, or whether travel, time off work, and cost were justified. These barriers were especially visible for county-town and rural participants, older adults, and employed participants with limited flexibility.

Vague recommendations were behaviorally weak. P07 said, ‘The doctor said to recheck, but the report did not say when. Recheck without a time is like no instruction; it lets me postpone and still feel that I obeyed' (female, 18–34 years, urban district, thyroid marker abnormality). P21 explained that his report remained in a drawer because the clinic required two buses and the correct department was unclear.

Online search sometimes substituted for triage because appointment systems required a department choice before professional advice. One participant described using Baidu, a major Chinese-language web search engine, as an informal triage tool: ‘I used Baidu first because the appointment system needed a department. The search result chose the department for me before a clinician did' (P04, male, 18–34 years, urban district, liver enzyme abnormality). By follow-up, P04 had used search and family discussion to narrow a possible route but had not obtained formal care; this was classified as family or online advice without formal care by follow-up.

Transport and family schedules shaped whether intention became service use. P30 had a plan but depended on her husband's availability to travel, whereas P21′s intention weakened when the correct department and a two-bus journey remained unresolved. These accounts illustrate why planned monitoring and delay or no action were analytically distinct.

Self-directed repeat testing formed a separate trajectory. P02 repeated liver-function testing because doing so was easier than choosing a doctor; the repeat result changed his appraisal but did not necessarily create interpretation. This recursive pattern is examined in the paired account below.

### Theme 5: Participants described communication as more actionable when explanations were respectful, proportionate, and time-bound

Participants wanted plain language without oversimplification. The most valued explanations addressed four linked questions: what the result might indicate, what it did not prove, when to repeat or consult, and which service was an appropriate entry point. This structure converted an undefined threat into a manageable task while preserving diagnostic uncertainty.

P35 described how a nurse explained HbA1c as a three-month average and wrote a time-bound repeat instruction: ‘It became a task, not a fear' (female, 35–49 years, urban district, glycemic abnormality). The interval was reported by the participant and is not presented here as a general clinical standard. P31 similarly wanted a translation ‘that did not make me childish,' emphasizing that clarity and dignity were linked.

Participants preferred concise prioritization and route information. P37 said, ‘Just tell me: common or uncommon, serious or not serious, and where to ask. It does not need to be long' (female, 18–34 years, urban district, liver enzyme abnormality). A non-blaming tone also mattered; P36 said that a statement such as ‘This is not an emergency, but it is not nothing' helped her remain calm enough to follow up.

Participants also wanted boundaries around uncertainty. P41 explained that a time-bound repeat instruction would have reduced midnight searching for kidney failure. Across cases, participants who reported clinician-linked follow-up commonly described a next step that combined timing and route, whereas delay/no action was often associated with concern without a route. These are qualitative associations, not evidence that the communication components caused clinically appropriate behavior.

### Paired longitudinal trajectories

Table 3 makes the distribution of dominant trajectories transparent, but paired narratives show the temporal mechanisms through which those categories were formed. The following cases were selected to illustrate contrasting transitions across the five themes, not because they were statistically representative of the follow-up subsample.

P43 began with recognition but little usable meaning. At baseline, she said, 'I did not understand the letters, but my son wrote: morning fasting, community clinic, next Tuesday. That was enough for me to go.' The written plan combined an interpretation she trusted with a date and a local service route. At the follow-up interview, she reported completing a community-clinic repeat test and receiving advice from a general practitioner. Her transition was therefore classified as clinician-linked follow-up. The analytic point was not that the study had established the clinical optimality of the interval; it was that uncertainty became navigable when meaning, timing, and route were assembled into one task.

P30 expressed an intention to seek follow-up at baseline, but that intention was conditional on transport and family scheduling. At the follow-up interview, she explained, ‘After the interview I still planned to go, but I waited for market day when my husband could drive. A plan without transport is not a plan.' She had retained concern and identified a feasible journey, but had not completed the visit by the interview date. This account was classified as planned monitoring rather than delay or no action because a time, transport arrangement, and intended service route were explicit. The study did not determine whether that timing was clinically optimal.

P02 followed a different route. At baseline, he described choosing a repeat liver-function test because repeating the test seemed easier than selecting a clinic. At follow-up, he said, ‘I repeated the liver function because it was easier than choosing a doctor. When the number was lower, I stopped thinking about why it was high'. The lower value reduced worry, but he still could not explain the initial abnormality or determine whether further professional review was needed. His trajectory was classified as self-directed repeat testing: new information fed back into risk appraisal and produced temporary reassurance, but it did not supply clinical context or a service-linked interpretation.

## Discussion

### Main interpretive claim

This study shows that non-emergent abnormal laboratory results did not become a navigable response simply because recipients were attentive or motivated. Reports and surrounding service systems often transferred interpretation, calibration, and navigation work to recipients and families. Participants noticed abnormality, searched, compared experiences, and intended to respond, yet the pathway remained fragile when priority, timing, or route was absent.

The distinction among actionability, action, clinical appropriateness, and outcome is central. Actionability describes whether communication creates the conditions for a proportionate, time-bound, navigable next step. Action is what a participant reports doing. Clinical appropriateness would require case-level review of values, symptoms, history, and recommendations, which this study did not perform. Outcome refers to what subsequently occurred. This distinction avoids treating all delay as failure or all completed service use as beneficial.

The longitudinal component revealed multiple reported trajectories rather than a binary action/non-action endpoint: clinician-linked follow-up, planned monitoring, family or online advice without formal care by follow-up, self-directed repeat testing, and delay or no action. New information from clinicians or repeat results could then revise meaning and risk, making the pathway recursive rather than a one-way sequence. Unresolved concern may also contribute to additional or potentially unnecessary service use, although the study did not classify this as a separate observed trajectory and did not adjudicate clinical necessity.

### Relationship to prior work

The findings support established health literacy models of access, understanding, appraisal, and application (1–3) while specifying where laboratory communication can fail. Prior work links limited health literacy with outcomes through access, communication, and self-care (6, 7), and Chinese clear-communication research highlights main messages, behavioral recommendations, numbers, and risk explanation (5). This study adds priority, temporality, and service entry as laboratory-result-specific conditions of application.

The results also extend patient-safety and laboratory-medicine research. Follow-up failures can contribute to missed or delayed diagnosis (14–17), while testing has value only when it informs decisions and avoids harm (18–20). Patient-portal and presentation studies show that direct access remains cognitively and emotionally demanding and that visual design can mislead (21–30). The present findings suggest that these difficulties can persist across paper and mobile reports and interact with service-navigation demands.

### Distributed interpretation, risk amplification, and navigation burden

Distributed health literacy helps explain why family involvement was neither simply assistance nor dependency. People drew on relatives' knowledge, hospital experience, transport, and appointment skills to seek, understand, and use information (43). In this study, distributed interpretation could translate a report into a plan, share uncertainty, normalize concern, or amplify fear. Recipient-controlled sharing is therefore more defensible than either excluding family from communication or automatically disclosing results to relatives.

The social amplification of risk framework clarifies how severe online possibilities, workplace comparison, and family narratives could amplify or attenuate concern as information passed through social and digital channels (44). Burden of Treatment Theory likewise helps explain why motivation did not erase the work of choosing a department, arranging transport, taking leave, paying for repeated services, and coordinating family support (45). The study's contribution is to connect these traditions within a laboratory-result pathway where meaning, risk, and navigation repeatedly interact.

### Implications in the era of direct-to-consumer laboratory testing

The findings also have implications for direct-to-consumer (DTC) laboratory testing, in which consumers initiate testing outside traditional clinician-mediated pathways. DTC services may improve convenience and autonomy but can separate the laboratory signal from test selection, clinical context, specimen quality, interpretation, confirmatory testing, and a clear follow-up route. Recent laboratory-medicine literature emphasizes that analytic accuracy is only one part of the total testing process and that consumer-initiated testing can create gaps at both the beginning and end of that process (46, 47).

The recursive model suggests that DTC reports may intensify salience without priority, meaning without context, concern without calibration, and recommendations without a route. Providers could therefore consider plain-language uncertainty, graded urgency, confirmatory-testing conditions, time-bound next steps, privacy information, and access to professional advice. Because the present participants received hospital-affiliated health examination reports rather than DTC tests, this is a conceptual extension rather than direct evidence and requires evaluation among DTC users.

### Conceptual contribution: a recursive result-to-action pathway

**Figure 1:**
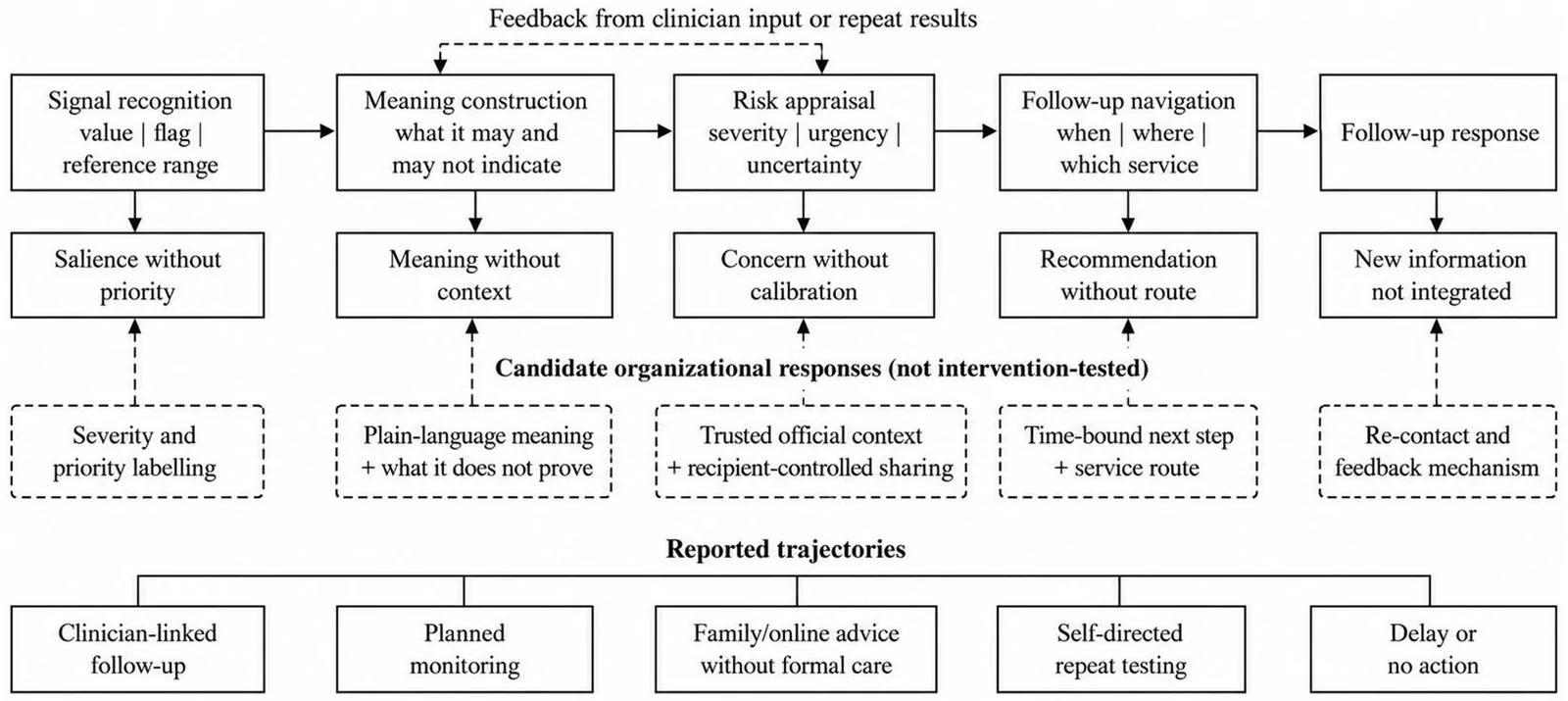
Recursive result-to-action model for the communication of non-emergent abnormal laboratory results. The top row represents the communication pathway from signal recognition to follow-up response. The second row shows unresolved failure points. Dashed boxes identify candidate organizational responses that were not intervention-tested and connect only to the corresponding failure points. From the follow-up response stage, the pathway branches to five parallel reported trajectories: clinician-linked follow-up, planned monitoring, family or online advice without formal care by follow-up, self-directed repeat testing, and delay or no action. These trajectories are descriptive categories rather than sequential stages or judgments of clinical appropriateness. A dashed feedback arrow indicates that clinician input or repeat results may revise meaning construction and risk appraisal. Cross-cutting contextual influences operate throughout the pathway, including age, education, rurality, family roles, digital context and navigation capacity, result type, and prior illness experience. Family and online information may recur across meaning construction, risk appraisal, and navigation.

The result-to-action model is shown in Figure 1. The communication pathway moves from signal recognition to meaning construction, proportional risk appraisal, navigation, and a follow-up response or trajectory. Beneath these stages are unresolved failure points and aligned candidate organizational responses. The candidate responses were not intervention-tested and are linked only to the corresponding failure points. From the final response stage, the model branches to five parallel reported trajectories rather than depicting them as sequential steps. A feedback arrow shows how clinician input or repeat results may revise meaning and risk.

The model adds specificity to broad access-understand-appraise-apply frameworks. It identifies salience without priority, meaning without context, concern without calibration, recommendation without route, and failure to integrate new information. Family and online information operate across stages rather than belonging to a single box, while age, education, rurality, family roles, digital access, result type, and prior illness experience act as cross-cutting contextual influences. The model is an empirically informed explanatory framework, not a prediction rule.

### Regional communication ecology

This regional two-center setting shaped the pathway through concrete mechanisms. Hospital applications and WeChat-linked mini-programs increased speed and visibility, but they did not necessarily add interpretive depth. Department-first registration pushed some participants toward Baidu or family advice. County-town and rural participants weighed tertiary-hospital trust against transport, work, cost, and the possibility of a local repeat test. Site A and Site B differed in release channels and handover practices, but the same five themes were present at both sites.

These mechanisms should not be generalized to all health examinations in China. They nevertheless resonate with broader tensions in health-system navigation during efforts to strengthen primary care and chronic disease prevention (48, 49). A recommendation such as ‘consult a specialist' may be navigable for an urban adult with flexible time and digital registration skills but insufficient for someone who must decide whether travel and time away from work are justified.

### Implications for public health education and health examination systems

The findings support an empirically informed set of candidate actionability components for laboratory-result communication. Table 4 organizes seven components: priority, meaning, uncertainty, timing, route, trusted support, and recipient-controlled sharing. These components are not claimed to be necessary, sufficient, or ranked, and they require clinical, usability, and implementation validation. The framework is not a clinical guideline and does not prescribe result-specific intervals or referral thresholds.

**Table 4:** Candidate actionability components for laboratory-result communication.

Component	Question the communication should answer
Priority	Does the finding call for prompt clinical review, routine follow-up, confirmation or repeat testing, monitoring, or lifestyle-focused action?
Meaning	What might this result indicate in plain language?
Uncertainty	What does the result not prove, and when might confirmation be needed?
Timing	When should the recipient repeat, monitor, or seek advice?
Route	What is the feasible entry point: community/primary care, repeat laboratory testing, or a named specialty?
Trusted support	Is there an official explanation or contact route that reduces reliance on uncontextualized search results?
Recipient-controlled sharing	Can the recipient choose a family-readable or shareable explanation while retaining control over disclosure?

These candidate components are empirically informed but are not asserted to be necessary, sufficient, or ranked. They require clinical, usability, and implementation validation. The table is not a clinical guideline and does not prescribe result-specific intervals or referral thresholds.

Health examination centers could test whether reports distinguish urgent from non-urgent priorities, explain what a result may and may not indicate, name an entry service, and provide a locally approved time window. Official report-linked information may reduce reliance on uncontextualized search results. Re-contact mechanisms could help integrate repeat results or clinician explanations back into meaning and risk appraisal rather than treating report release as the end of communication.

Family-facing design should remain under recipient control. Optional shareable explanations may help recipients who choose to involve relatives, while privacy, consent, and the recipient's authority over disclosure must be preserved. Digital design should also separate visual salience from urgency and should not assume that receiving an electronic report is equivalent to being able to navigate registration and follow-up.

### Equity implications

Actionability has equity implications because readability without a feasible route may create concern without access. County-town and rural participants described transport, time, cost, and uncertainty about tertiary-hospital necessity. Older adults sometimes relied on family translation, while electronic report access did not guarantee independent digital navigation.

Equity-oriented communication should therefore be assessed by both readability and navigability. A rural recipient may need to know whether a local repeat test is an acceptable first contact; an older adult may prefer an optional family-readable explanation; and a mobile report should pair color with priority and timing. Implementation studies should examine whether these features reduce or redistribute work across recipients, families, primary care, laboratories, and examination centers.

### Strengths and limitations

The study has several strengths: a report-anchored longitudinal design, purposeful variation, two sites, explicit site comparison, reflexive thematic analysis, framework matrices, complete quote audit, discrepant-case analysis, and feedback discussions. The transition matrix makes all 28 short-term trajectories visible without converting a purposive subsample into inferential statistics.

Several limitations require emphasis. First, the two centers were located in one prefecture-level region, limiting transferability to other provinces, private centers, primary care, DTC settings, and more highly digitalized systems. Second, participants who agreed to interviews may have been more reflective or concerned than those who declined. Third, follow-up behavior was self-reported and not verified through records or independently adjudicated for clinical appropriateness.

Fourth, the 28–43-day follow-up window captured short-term trajectories but may have classified clinically appropriate longer-interval monitoring as non-completion. Baseline interviewing may also have increased the salience of the abnormal result and influenced subsequent searching or action. Fifth, the study excluded results requiring emergency management, so the model should not be applied to critical-result notification. Sixth, the study did not assess whether the original tests were appropriately ordered, an important boundary for screening-related findings.

Seventh, baseline interviews combined own-report elicitation with vignettes, although final direct quotations were restricted to own-report or follow-up accounts. Eighth, interviews were conducted in person, by video, and by telephone; mode and occasional family presence may have shaped what participants disclosed. Interviews were limited to adults able to participate in Mandarin; the experiences of linguistic minorities and people with substantial visual, cognitive, or communication barriers may differ. Finally, rare result clusters were used only as analytic contrasts, and participant-level audit materials are not public because detailed combinations could increase re-identification risk.

## Conclusion

Non-emergent abnormal laboratory results are public health communication events, not self-interpreting data points. In this regional two-center study, recognition was insufficient without meaning, calibrated concern, timing, and a navigable route. The model separates communication actionability from reported action and from clinical appropriateness, and it shows how family, online information, clinician input, and repeat results can reshape the pathway over time. Health examination systems should test recipient-controlled, clinically reviewed communication components that support proportionate and navigable follow-up while preserving uncertainty and privacy.

## Data Availability

The datasets presented in this article are not readily available because the full interview transcripts, participant-level master record, and complete quote audit contain combinations of health findings, residence, family circumstances, and service-use trajectories that could increase re-identification risk in this regional two-center sample. De-identified interview guides, coding definitions, aggregate matrices, and selected excerpts may be made available to editors or qualified researchers through controlled access upon reasonable request, subject to ethics approval, institutional permission, and participant consent conditions. Requests to access the datasets should be directed to Lei He, 309999806@qq.com.
